# Report of a Case of Disarticulation of the Hip for Sarcoma of the Femur, with Remarks upon the Diagnosis and Prognosis in Sarcoma of the Femur*Read before the Western Surgical and Gynecological Society, at St. Joseph, Mo., December 29, 1902.

**Published:** 1903-02

**Authors:** A. E. Halstead

**Affiliations:** Chicago, Professor of Surgery, Chicago Polyclinic; Attending Surgeon, Cook County and Chicago Baptist Hospitals; Consulting Surgeon, Illinois Charitable Eye and Ear Infirmary


### TEXAS MEDICAL JOURNAL.

ESTABLISHED JULY, 1885.

PUBLISHED MONTHLY.—SUBSCRIPTION $1.00 A YEAR.

VOL. XVIII. AUSTIN, FEBRUARY, 1903. No. 8.

### Original Contributions.

For Texas Medical Journal.

### Report of a Case of Disarticulation of the Hip for Sarcoma of the Femur, With Remarks Upon the Diagnosis and Prognosis in Sarcoma of the Femur.*

BY A. E. HALSTEAD, M. D., CHICAGO,

Professor of Surgery, Chicago Polyclinic; Attending Surgeon, Cook County and Chicago Baptist Hospitals; Consulting Surgeon, Illinois Charitable Eye and Ear Infirmary.

Miss Katherine G., American, age 21, presented herself at the Chicago Polyclinic in April, 1899, suffering from a painful swelling of the bone above the left knee joint.

She gave the following history: Family history negative; has never had any serious illness; no history of any venereal infection. The present trouble began after falling from a carriage in June, 1898. Immediately after this fall she had pain in the knee, accompanied by swelling and stiffness of the joint. After a few days rest the pain subsided and she again was able to be about. Later, in August of the same year, she again experienced pain about the knee; this was of a neuralgic character, and was not at first constant. It soon became very severe and continued without interruption, being generally more severe at night. By the middle of September she was forced to keep her bed because of the great pain

*Read before the Western Surgical and Gynecological Society, at St. Joseph, Mo., December 29, 1902.

that was caused by any movement of the limb. At this time she first consulted a physician, who recommended rest and the application of a plaster of Paris cast. This treatment was carried out, but only seemed to make the pain more severe. After a few weeks, the cast was removed, but was later reapplied. When she consulted me she was wearing a cast, the case being considered one of tuberculosis of the knee.

During the last few months preceding her entrance into the hospital she had steadily lost flesh. Her appetite was poor and her rest was broken by the severe pain she suffered. During the whole course of the disease her temperature had been above normal, varying from 99 to 102 degrees.

Physical examination, at the time of entrance, showed the patient to be greatly emaciated, pale and extremely nervous. The chest and abdomen were apparently normal. Examination of the urine, negative; the muscles of the left lower extremity were markedly atrophied. The left knee and lower third of the thigh were apparently larger than the right. On measurement, the left femur, just above the condyles, was found to be 4 c. m. larger in circumference than the right. The left knee joint, measured across the center of the patella, was slightly larger than the right. There was a uniform enlargement of the lower end of the left femur that was regular in outline, and exceedingly sensitive on pressure. The knee was fixed with the leg flexed at an angle of 45 degrees. Any attempt at movement of the joint caused great pain. Under anesthesia, the leg could be fully extended. No limitation of movements in any direction was present. Nothing abnormal about the joint was found. Puncture of the joint was made, but no fluid obtained. Upon the negative findings in the joint and the peculiar enlargement of the femur a diagnosis of the sarcoma of the femur was made. Through a small incision made while the patient was under the anesthetic, a piece of the tumor was excised, and subjected to a microscopic examination. It proved to be a peripheral ossifying spindle-celled sarcoma.

Upon recognizing the nature of the disease, amputation at the hip-joint was recommended. This was performed on the 20th of May, 1899, the method of Wyeth for the control of hemorrhage being employed. The patient suffered but little shock from the operation, and made a rapid and satisfactory recovery from the operation. The wound healing throughout by primary union. At the time of the operation, the inguinal and iliac glands were found enlarged, and were removed. Microscopic examination did not, however, show any evidence of carcinomatous change in them. The patient's general health rapidly improved after this operation, and she was able to leave the hospital at the end of three weeks. From that time until the present, three years and seven months after the operation, her health has continued good and there is no evidence of a return of the disease. At the time she left the hospital, her weight was 98 pounds, at the present time it is 180 pounds. The tumor was examined by Dr. Paul F. Morf, whose report upon its histologic structure is as follows:

"Microscopically, the tumor is seen to be mainly made up of round or more or less polyhedral cells which resemble young hyperplastic periosteal cells in appearance. Some which have apparently reached a more mature stage resemble young cartilage cells. The former are for the most part closely bunched together, and vary in size from two to three times the size of a red blood corpuscle. The protoplasm is not abundant, and the nuclei are separated by a small amount of coarsely fibrous intercellular substance, and where this intercellular substance becomes more abundant, the tumor cells tend to resemble cartilage cells more in structure. They become more circular in outline, the protoplasm is more abundant and the nuclei relatively smaller. Yet in other parts, there are dense bands of fibrous tissue, so the tumor tissue has more the appearance of periosteum. Scattered through the tumor tissue are islets of irregular outline, which, in the gross specimen, contained lime salts, and which had to be decalcified in order that the tissue could be cut. These areas stain deeply with hematoxylin, and are made up of a homogeneous intercellular substance containing large round cells with a large amount of protoplasm and a small nucleus, the cells themselves surrounded by a capsule. These islets have an irregular dentate and branched outline, and have apparently developed from the areas just described where the intercellular substance is abundant. In a very few places, there are islets which resemble true bone, the decalcified intercellular substance being arranged in lamelle, and between these remain a few small shrunken stellate cells, resembling bone cells, and lying in small lamelle like those of normal bone. The blood vessels are very few in number, their walls, as is usual in sarcomas, are made up of tumor cells."

Before discussing the diagnosis and prognosis of sarcoma of the femur, it is essential that we have a clear idea of the origin and histologic structure of these tumors.

According to their point of origin from the bone, they may be grouped into central, myelogenic or intraosseous and peripheral or periosteal.

The first group has its starting point from the osteoblastic layer lining the medullary canal, and is usually a giant-celled or myeloid tumor. Besides these, giant-celled sarcomas, spindle or round-celled sarcomas may also originate from the medullary tissue. These, however, are not common, particularly the pure spindle and round-celled tumors, but mixed tumors with a preponderance of giant cells are quite common.

The peripheral or periosteal tumors proceéd from the layer of osteoblasts or embryonic cells that separate the periosteum from the bone.

These tumors are mostly spindle-celled sarcoma, while occasionally we find round-celled or mixed round and giant-celled tumors. By far the greatest number are spindle-celled or contain a large proportion of spindle cells.

Concerning the relative frequence of these different forms of sarcoma, Gross found, in reviewing one hundred and sixty-five cases of sarcoma of long bones, that seventy or 42 per cent. were giant-celled tumors; forty-five were spindle-celled osteoid sarcoma; sixteen central spindle-celled; thirteen periosteal round-celled; twelve central round-celled, and nine pure periosteal spindle-celled sarcomas.

Of these, 75 per cent. of the peripheral involved the lower epiphysis of the femur. There were seventy central giant-celled tumors of which nineteen or 27.01 per cent. involved the lower and two the upper extremity of the femur.

Central giant-celled or myeloid sarcoma are found principally in the lower epiphysis, and occasionally in the upper. They are of rather slow growth and represent the lowest degree of malignancy of any of the bone sarcomas. A few, no doubt, are benign and correspond to the class of tumors described by Bland Sutton as myelomata, and considered by him as being essentially benign.

Many of the earlier writers considered all pure myeloid tumors as innocent. Among those who regarded only the mixed tumor as malignant, are Nellaton, Wilks and Förster. It must be emphasized that in none do we find only giant cells, but all "mixed" tumors containing spindle and round cells and varying propositions. From a clinical standpoint, therefore, it is never safe to consider any of them innocent.

Undoubtedly, the degree of malignancy varies greatly in individual cases. The rapidly growing vascular and particularly the osteoid sarcomas of this type possess a great degree of malignancy as is evidenced by local infection of surrounding tissue. lymphatic involvement and metastasis in distant organs. Invasion of the neighboring tissues occurs only when the tumor has passed its bony confines. This may take years. As a rule, these tumors are confined to the parts from which they originate for a considerable time. During this period removal of the tumor alone is often sufficient to effect a cure.

Lymphatic involvement is the exception. In quite a considerable number of cases the inguinal and femoral glands are enlarged, but, as a rule, no sarcomatous change is found in them on microscopic examination. Metastases occur late in myeloid tumors. In the calcifying and ossifying giant-celled sarcoma, metastasis occurred in 58 per cent. of the cases reviewed by Gross.

The clinical picture presented in giant-celled sarcoma is usually that of the slow growing regular, spherical, more or less firm tumor involving one of the extremities, usually the lower of the femur. The tumor mass is incased in a bony capsule for some time. As the capsule becomes thinned, crepitation on the slightest pressure is observed. In extremely vascular tumors, pulsation may be a prominent feature. Even in cases where no great enlargement of the vessels is found, pulsations may be an early symptom.

On section, the tumor appears as a soft mass of deep red color, having the consistency of muscle tissue. Occasionally the color varies from light brown to yellow, depending upon the amount of fibrous tissue and upon the degenerative changes present. In vascular tumors extensive blood cysts may be found. In the calcified and ossifying tumors, hard masses and sharp bony spicules can be seen and felt on the cut surface. The skin covering these growths usually remains unchanged. In but few are the subcutaneous veins enlarged or the surface ulcerated. Their growth is usually progressive. Occasionally, it is temporarily arrested, as in the case recorded by Hutchinson, in which a giant-celled sarcoma, after having reached the size of a child's head in fourteen months, ceased to develop for four years, when, without any apparent cause, it rapidly grew until a fatal termination ensued. Certain conditions, such as trauma, pregnancy and laceration seem to stimulate them to increased activity. Pain, though not a constant symptom, usually is present. Gross states that it is a prominent feature in about 50 per cent. of the cases. In the majority of cases, the neighboring joint is not involved until very late in the disease, the articular certilage offering considerable resistance to the growth of the tumor into the joint.

Fracture of the bone through the tumor occurs frequently and often first directs the patient to a surgeon. These tumors occur more frequently in the male than in the female, and are most common in young adults. In childhood, the disease is rare.

Central spindle-celled tumors are next to the giant-celled, the most frequent. Myelogenic tumors of long bones, although they occur in one-third of the cases in the lower extremity of the femur, are found in the shaft relatively more frequent that are giant-celled tumors. They posses a higher degree of malignancy than do the giant-celled myeloid sarcoma, and occur as smooth or nodulated tumors, and are usually limited by a bony or fibrous capsule. Pain is nearly a constant symptom of these tumors. Spontaneous fracture or fracture from slight causes occurs in about one-half of the cases. Metastasis occurs in a large proportion while lymphatic involvement has never been observed.

Central round-celled sarcomas invade the shaft, and the epiphysis of the femur with equal frequency. The are the most rapidly growing tumor of bone, possessing a high degree of malignancy. They are usually vascular and rapidly undergo the retrograde changes common to all sarcoma, mucoid degeneration being the most common. Early in their development they break through the cortical layer of bone surrounding them, and quickly invade the neighboring soft tissues. Lymphatic involvement and generalization of the growth in distant organs occurs in a relatively short time. The growth of these tumors is constantly associated with great pain, rapid emaciation, cachexia and early death. Hemorrhages into the tumor, ulceration of the skin and spontaneous fracture occur more frequently than in any other variety of bone sarcoma.

Peripheral or periosteal sarcoma originate in the soft osteogenic layer of the periosteum, and in reality develop between the bone and periosteum. They are limited by the dense layer of the periosteum, which for some time offers sufficient resistance to prevent invasion of neighboring tissue. Histologically, they are either round or spindle-celled sarcoma. Giant-cells where found, probably reach the peripheral growth by extension through the bone from the medullary canal. Both spindle and round-celled periosteal growths exhibit a great tendency to ossification and calcification. According to Gross, 67 per cent. are osteoid sarcoma.

In general, peripheral sarcoma differs from central, in that they are not so common and that they are decidedly more malignant. Again they occur earlier in life and are more generally painful than the central tumors. Pulsation, which occurs in 20 per cent. of the central tumor, is seldom observed in peripheral tumors. Spindle and round-celled peripheral sarcoma usually involve the lower epiphysis of the femur. The spindle-celled tumors grow steadily, but as a rule, slowly. Local infection of the surrounding tissue and neighboring glands occurs late. The skin is frequently ulcerated. Joint invasion is the exception.

In the ossifying or osteoid spindle-celled tumor, metastasis in the distant organs, particularly in the lungs, is exceedingly common, occurring more often than in any other variety of bone sarcoma. Generalization of these tumors occurs early, generally within a few months after the tumor begins to grow.

The diagnosis of sarcoma of the femur, and especially in the early stage of the disease, is often very difficult. In all varieties of sarcomas of the femur, as in other malignant growths, there is a time when the disease is distinctly local. It is in this stage that long local treatment, if it is to be successful, must be instituted. If the true character of the disease is not recognized until generalization has taken place, no treatment known at the present time, can in any way favorably influence its progress.

The most constant symptoms of sarcoma of the femur are pain, enlargement of the bone and spontaneous fracture or fracture from slight trauma.

Pain is the earliest symptom in most forms of sarcoma of the bone. In the periosteal, particularly in the rapidly grown tumors, it is constant and is the first indication of the onset of the disease. Taking all the different forms of sarcoma, it is present in about 85 per cent. of the cases, and is continuous throughout the course of the disease, varying in character and intensity, being most severe in periosteal and less constant, usually less intense in the myelogenic tumors.

In central tumors, particularly in those of slow growth, it is frequently of a neuralgic character, and not always constant, but of an intermittent character. Thickening of the bone is, of course, always a symptom. In many of the slow growing central tumors, considerable time may elapse before the enlargement can be detected. This is often the case when the disease involves the upper epiphysis of the tumor. In periosteal growths, particularly of the diaphysis of the bone, the tumor can readily be recognized.

In central tumors, the bone is uniformly enlarged throughout its whole circumference; usually the thickened part is smooth and regular in outline. As the disease progresses, the capsule of bone enclosing the tumor becomes thinned so that the slightest pressure will break the thin laminated covering and give distinct crepitation.

Perioesteal sarcoma, as a rule, involves the whole circumference of the bone. When the epiphysis is the seat of disease, the extremity of the bone is uniformly enlarged. Rarely, we find circumscribed growths projecting from one surface. When the shaft is involved, the enlargement is spindle-shaped. When the tumor has broken through the periosteum, the growth becomes extremely irregular and nodular.

In the vascular central tumors, absorption of the bone may take place rapidly without occasioning much pain; in such a case, pathologic fracture may first direct the patient to a surgeon. In central tumors spontaneous fracture or fracture from slight injury occurs in about 60 per cent. of the cases. The early occurrence of such a fracture favorably influences the prognosis of the disease.

In central round-celled sarcoma of the shaft, spontaneous fracture occurs more frequently than in any other variety of bone sarcoma. In peripheral tumors, particularly in spindle-celled and osteoid sarcomas, spontaneous fracture seldom occurs. Invasion of the neighboring lymphatics occurs most frequently in peripheral and round-celled tumors. In spindle-celled peripheral growths it is rarely that we find the glands enlarged. In osteoid tumors, grandular involvement, as well as metastasis by way of the blood vessels, occurs early and is nearly a constant finding. It may be well to call attention to the fact that hyperplasia of the glands without there being any sarcomatous invasion of the organ, is quite common. Lymphatic involvement occurred in five of the forty-four cases collected by Nasse. Of these three were periosteal and two myelogenic tumors. Lœffler states that lymphatic invasion occurs in 13 per cent. of all cases of peripheral sarcoma. In all cases where the lymphatics are involved, the prognosis is extremely bad. In many cases of sarcoma, particularly rapidly growing vascular tumors, an elevation of the body temperature is constant, varying from 99 to 102 degrees. In some it is only present after exercising or when the tumor has been subjected to trauma.

The cause of this elevation of temperature is probably due to the absorption of toxines, resulting from degenerative changes occurring in the tumor, or from absorption of the blood from the blood cysts within the growth. Usually in these cases, where absorption of blood is the cause of the temperature, rest in bed with cold applications to the diseased part will cause the temperature to drop. In soft tumors, particularly those undergoing retrograde changes, the temperature is constant and continued throughout the entire course of the disease.

Of late years a considerable number of writers have directed attention to the diagnostic significance of persistent albumosuria in bone tumors. At the present time we know that albumose is found in the urine in a great variety of pathologic conditions, chief among which may be mentioned chronic suppuration in any part of the body. It is also quite a constant finding in osteomalacia and in certain tumors of bone, particularly enchondromata and myelomata. It can usually be found in the urine in bone sarcoma but can not be used as a means of differentiating malignant from benign tumors, nor chronic infections of bone from new growths.

It is well known that the prognosis in bone sarcoma is generally bad. In sarcoma of the femur, 70 per cent. of the periosteal are the osteoid variety, which are among the most malignant of all bone sarcoma.

In the central giant-celled tumors, the prognosis is relatively good. In a few, the disease seems to be essentially benign. It has been generally held that freedom from recurrence for three years should be considered as a cure. This is farther from the truth in sarcoma of the bone than in other malignant tumors. Local recurrences have occurred as late as twelve years after amputation, while in one case of central sarcoma lung metastases were first evident twenty-five years after the tumor began to develop.

Max Bussey reports a case of death from lung metastasis four and one-half years after disarticulation of the hip. In a case of hip-joint amputation for sarcoma of the femur operated on by McGraw, metastasis upon the shoulder occurred thirteen years after the amputation. Borck collected from the literature, 111 cases of disarticulation of the hip for sarcoma of the femur operated on in European clinics, of which 87 survived the operation. Of these, not one could be said to have been cured. In a recent paper, Jenckel was able to collect ten cases of recovery after operation for sacoma of the femur. He reports thirty-five cases, nineteen of which are from the clinic of Koenig (1880-1895) and sixteen from Braun's clinic, operated on between the years 1884-1901. Of these thirty-five cases four should be considered cured. Of these four recoveries, three were treated by amputation of the thigh and one by disarticulation of the hip. In the literature he was able to find six recoveries recorded. These cases were operated on by Holmes, Rose (two cases), Kramer, Weisenger and Mikulicz. The length of time that had elapsed after the operation was respectively 8, 5, 16, 12, 6, and 4½ years.

In the four cases of record reported by Jenckel from the clinics of Koenig and Braun, the patients had survived the operation, 15½,

6¾, 15 and 12¾ years. Of these ten cases, seven were central and three periosteal growths.

REFERENCES.

1. *Borck:* Ueber die Heilbarkeit Maligner Neubildungen des Oberschenkelknochens durch die Exarticulation der Unteren Extremitat im Hüftgelenke.—Langenbeck's Archiv. Bd. 40-941.

2. *Bussey, Max:* Exarticulation Coxæ wege Sarkome des Oberschenkels.—Inaug. Diss. Greifsnald, 1897.

3. *Gross:* American Journal Medical Sciences, 1879.

4. *Jenckel:* Beitrag zur Kenntness der Knochensarkome des Oberschenkel.—Deutsch Zeitschrift für Chirurgie Bd. 61, p. 66.

5. *Luttig:* Ueber Exarticulation im Hüftgelenkwegen Maligner Neubildungen am Femur.—Inaug. Diss. Marburg, 1891.

6. *Löffler:* Zur Prognose Knochensarkome.—Inaug. Diss. Erlangen, 1896.

7. *Nasse:* Die Sarkome der langen Extremitatenknochen.—Langenbeck's Archiv. Bd. 39, p. 886.

